# Long‐term outcomes in patients with advanced adrenocortical carcinoma after image‐guided locoregional ablation or embolization

**DOI:** 10.1002/cam4.3740

**Published:** 2021-03-09

**Authors:** Michal Mauda‐Havakuk, Elizabeth Levin, Elliot B. Levy, Venkatesh P. Krishnasamy, Victoria Anderson, Nidhi Jain, Hayet Amalou, Tito Fojo, Maureen Edgerly, Paul G. Wakim, Marybeth S. Hughes, Jaydira del Rivero, Bradford J. Wood

**Affiliations:** ^1^ Center for Interventional Oncology, Radiology and Imaging Sciences Clinical Center, and the National Institute of Biomedical Imaging and Bioengineering National Institutes of Health Bethesda MD USA; ^2^ Center for Interventional Oncology, Radiology and Imaging Sciences Clinical Center National Institutes of Health Bethesda MD USA; ^3^ Division of Medical Oncology Department of Medicine Columbia University Medical Center New York NY USA; ^4^ Office of Research Nursing Center for Cancer Research National Cancer Institute National Institutes of Health Bethesda MD USA; ^5^ Biostatistics and Clinical Epidemiology Service National Institutes of Health Clinical Center Bethesda MD USA; ^6^ Surgical Oncology Eastern Virginia Medical School Norfolk VA USA; ^7^ Developmental Therapeutics Branch Medical Oncology Service National Cancer Institute National Institutes of Health Bethesda MD USA; ^8^ Center for Interventional Oncology, Radiology and Imaging Sciences Clinical Center National Institute of Biomedical Imaging and Bioengineering and National Cancer Institute Center for Cancer Research National Institutes of Health Bethesda MD USA

**Keywords:** adrenocortical carcinoma, combinational therapy, endocrine tumors, interventional oncology, locoregional therapy

## Abstract

**Background:**

To evaluate outcomes and survival rates in patients with metastatic adrenocortical carcinoma (ACC) who were treated with image‐guided locoregional treatments (IGLTs).

**Purpose:**

To evaluate the overall survival (OS) and clinical impact of IGLT in the management of patients with advanced metastatic ACC.

**Methods:**

Retrospective review of 39 patients treated with IGLT between 1999 and 2018 was performed. Short‐ and long‐term efficacy of treatments were defined based upon imaging and clinical data. Subgroup survival analysis was performed on patients with metastatic disease at diagnosis (N = 17) and compared with the same stage group from the most recent National Cancer Database (NCDB) report. Statistical analysis was performed using Cox proportional hazards model.

**Results:**

Treatments were performed at different anatomic sites including liver (N = 46), lung (N = 14), retroperitoneum (N = 5), bone (N = 4), subcutaneous (N = 2), and intracaval (N = 1). Radiofrequency, microwave, cryoablation, or a combination of two modalities (45, 18, 3, 3, respectively) were used in 69 ablation sessions. Intra‐arterial procedures were performed in 12 patients in 18 treatment cycles (range 1–3 per patient). As of a 2019 analysis, 11 patients were alive with a mean follow‐up of 169 months (range 63–292 months) from diagnosis. Two‐ and 5‐year OS rates for all patients were 84.5% and 51%, respectively, and 76.5% and 59% for patients with metastatic disease at diagnosis (N = 17). This compares favorably with an NCDB report of 35% 5‐year survival rate for patients with metastatic disease. Female gender and longer time from diagnosis to first IGLT were found to be predictors of prolonged survival with hazard ratios of 0.23 (*p* < 0.001) and 0.66 (*p* = 0.001), respectively.

**Conclusion:**

IGLT may be associated with prolonged life expectancy in select patients with metastatic ACC.

## Graphical abstract

Treatment of patients with advanced adrenocortical carcinoma with image‐guided locoregional therapies may be associated with prolonged survival, especially in females. Challenging clinical decisions for this rare disease with few effective treatment options may be informed by our two‐decade experience with locoregional therapies. Such a combined approach with surgery, ablation, and/or embolization may be associated with better than previously reported long‐term patient survivals.

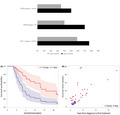

## 1 INTRODUCTION

Adrenocortical carcinoma (ACC) is a rare, heterogeneous, and aggressive disease, with at least 50% of patients having metastases at initial presentation, and has no broadly effective treatment option other than repeated surgical resection.^1^, ^2^, ^3^, ^4^ The prognosis of ACC is grim and depends on the disease stage at presentation, 5‐year survival is 60%–80% for tumors confined to the adrenal space, 35%–50% for locally advanced disease, and much lower in case of metastatic disease with reported percentages ranging from 0% to 28%.^5^

Diverse approaches to ACC are often considered standard of care, although these are often supported by anecdotal and less than high‐level evidence. Systemic therapies have limited efficacy, with no data on the role if any of adjuvant chemotherapy. Mitotane is currently the only drug approved for ACC, although its low efficacy and risk to benefit ratio remain topics of debate.^6^ Only two randomized controlled trials on advanced ACC have been reported in recent English literature.^7^, ^8^ Common combination regimens based on the FIRM‐ACT trial^7^ are etoposide, doxorubicin, and cisplatin (EDP) plus mitotane, or streptozocin plus mitotane. EDP plus mitotane had a significantly better response rate (23.2% vs. 9.2%) and progression‐free survival (5.0 months vs. 2.1 months) than streptozocin plus mitotane. However, overall survival (OS) rates were dismal, regardless of choice of systemic combination. Additionally, these chemotherapies carry side effects.

The only potentially curative therapy is complete surgical resection.^9^, ^10^ However, due to the extent of disease at presentation and/or contraindications to surgery, complete surgical resection is not an option in many patients. Even when complete radical surgical resection is achieved, up to 80% of patients experience recurrence.^10^ Some patients with ACC undergo repeated resections, with inherent morbidity and technical challenges due to adhesions, disease distribution, and adjacent anatomy. More recently, image‐guided locoregional treatments (IGLTs) have been increasingly employed, usually at later stages of disease recurrence or progression. These include thermal ablation (radiofrequency, microwave, and cryoablation) and transarterial embolization, chemoembolization, or radioembolization. Initial experience with these IGLTs has been promising, but with unknown long‐term efficacy or survival data.^11^, ^12^, ^13^

This retrospective, single‐center, several decades report aims to evaluate the OS and clinical impact of IGLT in the management of patients with advanced metastatic ACC.

## 2 METHODS

### 2.1 Data collection and clinical assessment

All patients (N = 39) were consented and enrolled in a number of Institutional Review Board (IRB)‐approved investigational studies of ACC at a single tertiary referral center, including an IRB‐approved retrospective reporting protocol that met criteria for waiver of further consent. Retrospective review of records identified all patients diagnosed with metastatic ACC who underwent IGLTs at our institution between 1999 and 2018. Data were collected and image review performed from January to April 2019, survival data were collected in August 2019, and statistical analysis was then performed. A small number of these patients (N = 8) have been included in prior publications on shorter term outcomes following ablation of ACC, with longest follow‐up of 3 years.^11^, ^13^, ^14^ Inclusion criteria required patients with pathology‐proven ACC, from a primary or a metastatic tumor resection or from percutaneous biopsy, which was obtained at the study site and a variety of institutions. Before local treatments were offered, patients were reviewed in a multidisciplinary team tumor board, typically including medical oncologists, surgeons, radiation oncologists, pathologists, diagnostic radiologists, and interventional radiologists with expertise in interventional oncology. Disease staging was performed using history, physical examination, labs, pathology, and imaging. Inclusion criteria also included patients with progressive, metastatic ACC that was unresponsive to systemic therapy and patients who were determined to have unresectable disease, were poor surgical candidates, or had rapidly growing foci of disease that were deemed safely targetable with IGLT by the multidisciplinary team. Spread of disease to multiple organs was not a contraindication to treatment of organ‐dominant sites if the other metastases were not considered acutely survival‐limiting.

All patients were treated with at least one of the following IGLTs: radiofrequency ablation (RFA), microwave ablation (MWA), cryoablation (CRYO), transarterial chemoembolization,^15^ or bland embolization (TAE). IGLTs were only applied to advanced disease as secondary, tertiary, or palliative therapy, and were never used for initial treatment of primary tumor. The number, type, anatomic location, outcome, imaging results, anesthesia type, adjunctive techniques of fusion guidance, and hydrodissection, duration of follow‐up, and complications of each treatment were recorded.

### 2.2 Technical efficacy assessment

All available pretreatment and follow‐up imaging were initially interpreted by at least two board‐certified radiologists, and subsequently reviewed by a third board‐certified radiologist. All imaging was then re‐reviewed for agreement by an additional board‐certified interventional radiologist. Equivocal imaging was defined as imaging with discrepancy in interpretation or lacking definitive evidence of local failure or local technical success. Such cases were resolved by panel consensus of three radiologists. The most recent follow‐up imaging ranged from 1 month to 194 months posttreatment. The term “session” referred to a single intervention event, either ablation or embolization. Percutaneous ablation “session” referred to a single intervention event that consisted of one or more ablations performed on one or more tumors. Each ablated tumor was individually assessed for response. Efficacy was concluded if there was a lack of enhancement, lack of avidity on PET imaging, and/or no growth on follow‐up imaging. These imaging features were used to classify tumors as completely or incompletely ablated. For transarterial interventions, multiple sessions at the same anatomic region were defined as one “cycle”. Efficacy assessment for each cycle of transarterial treatment was concluded if there was lack of contrast enhancement on CT and/or MRI, lack of avidity on PET imaging, and/or no tumor growth on follow‐up imaging, for each anatomic region. All clinic notes, prospective imaging reports, retrospective imaging reviews, and available follow‐ups were reviewed and classified as successful or unsuccessful for each cycle or session according to standard guidelines.^16^, ^17^

### 2.3 Statistical analysis

Cox proportional hazards regression analyses were used for data analysis. OS was calculated from the date of initial diagnosis until the date of death or, for censored data, until August 2019, at which time all living patients had achieved 5‐year OS. In order to compare survival rates in this report to previously reported survival rates, we performed subgroup survival analysis. Seventeen patients with stage IV disease at diagnosis were identified and survival rates from diagnosis were compared with the same stage group from the most recent National Cancer Database (NCDB) report.^18^ A stepwise approach was used to select important predictors of survival time among the following 14 candidate variables: age at diagnosis, patient gender, tumor stage at diagnosis, treatment with mitotane, any treatment with systemic chemotherapy (other than mitotane), treatment with multiple IGLT modalities, surgical resection of metastatic tumor(s) following the initial surgery, time from initial diagnosis to first IGLT, number of ablation sessions per patient, number of transarterial cycles per patient, total number of interventions per patient (ablation sessions + transarterial cycles), two proportions of tumors that were completely ablated (completely ablated tumors/total tumors treated, and completely ablated/total ablation sessions), proportion of successful transarterial cycles (successful transarterial cycles/total transarterial cycles), and proportion of successful treatments of both categories (completely ablated tumors + successful transarterial cycles)/(ablated tumors treated +transarterial cycles). A *p*‐value cut‐off for a variable to “enter” the model was set at 0.1, and to “stay” in the model set at 0.01. Kaplan‐Meier survival curves were generated based on the final model. The SAS statistical software (SAS Institute) was used for this analysis.

## 3 RESULTS

Thirty‐nine patients with metastatic ACC were included in this analysis. The median age at diagnosis was 44.5 years, with an age range of 22–67 years. Twenty‐three of the patients were female (59%). Seventeen patients had stage IV disease at diagnosis (43.5%). All but two patients received mitotane (95%); all but three received additional systemic therapy (92%). All patients underwent surgery prior to any local interventional procedure. Twenty‐eight of the patients (72%) had at least one surgery in addition to the primary resection.

Patients had IGLTs for metastatic tumors at a variety of time points and anatomic locations. Thirty‐five patients had percutaneous thermal ablations in a total of 69 treatment sessions. Treatment sessions per ablation modality were as follows: 45 RFA, 18 MWA, three CRYO, and three treatment sessions in which two different ablation modalities were used. Five RFA sessions were performed during heat‐deployed liposomal doxorubicin infusion.^19^ Nine tumors were treated multiple times (eight tumors were treated twice; one tumor was treated four times). A range of 1–10 sessions per patient, with mean of 1.97, were performed. In each session, one to three tumors were treated. A summary of patient characteristics and treatment anatomic sites is provided in Table 1. Technical efficacy of ablation was defined on follow‐up imaging per standard criteria. Interval and type of follow‐up imaging varied by patient, but generally included CT (and/or MRI and/or PET CT) imaging within 3 months post IGLT, followed by interval imaging, as clinically indicated. A total of 84 tumors were ablated. Technical efficacy of complete ablation was achieved successfully in 52 (62%) of the tumors. There was no imaging follow‐up after one tumor ablation, thus completion assessment could not be performed; this was classified as not complete.

**TABLE 1:** Patient characteristics and treatment locations

	Average (range)
Age at Dx (years)	45 (20–67)
Number of ablations (in ablation patients N = 35)	2 (1–10)

	Count (percent)
Gender	
Female	23 (59.0)
Male	16 (41.0)
Race	
White	35 (90.0)
Asian	2 (5.0)
Black	1 (2.5)
Hispanic	1 (2.5)
ECOG	
0	10 (25.7)
1	26 (66.7)
2	1 (2.5)
3	1 (2.5)
Unknown	1 (2.5)
Steroid secretion	
Yes	6 (15.4)
Yes, based on symptoms	9 (23.0)
No	11 (28.2)
Unknown	13 (33.3)
Metastasis at Dx	
Yes	17 (43.5)
No	22 (56.5)
Mitotane TRX	
Yes	37 (95.0)
No	2 (5.0)
Chemotherapy TRX	
Yes	36 (92.5)
No	3 (7.5)
Surgery later then primary resection	
Yes	28 (71.8)
No	11 (28.2)
IGLT TRX site per procedure	
Liver	46 (64.0)
Lung	14 (19.5)
Retroperitoneum	5 (7.0)
Bone	4 (5.5)
Subcutaneous	2 (2.7)
Intracaval	1 (1.3)

Abbreviations: Dx, diagnosis; TRX, treatment.

Twelve patients were treated with 35 transarterial treatment sessions in 18 treatment cycles. Transarterial treatments included bland embolization (N = 5) and doxorubicin‐eluting bead embolization (N = 30). A median of 2.5 and mean of 2.9 sessions per patient were performed, with a range of 1–7 sessions per patient. Eight patients treated with both ablation and transarterial treatments are included in the aforementioned patient numbers.

**FIGURE 1:**
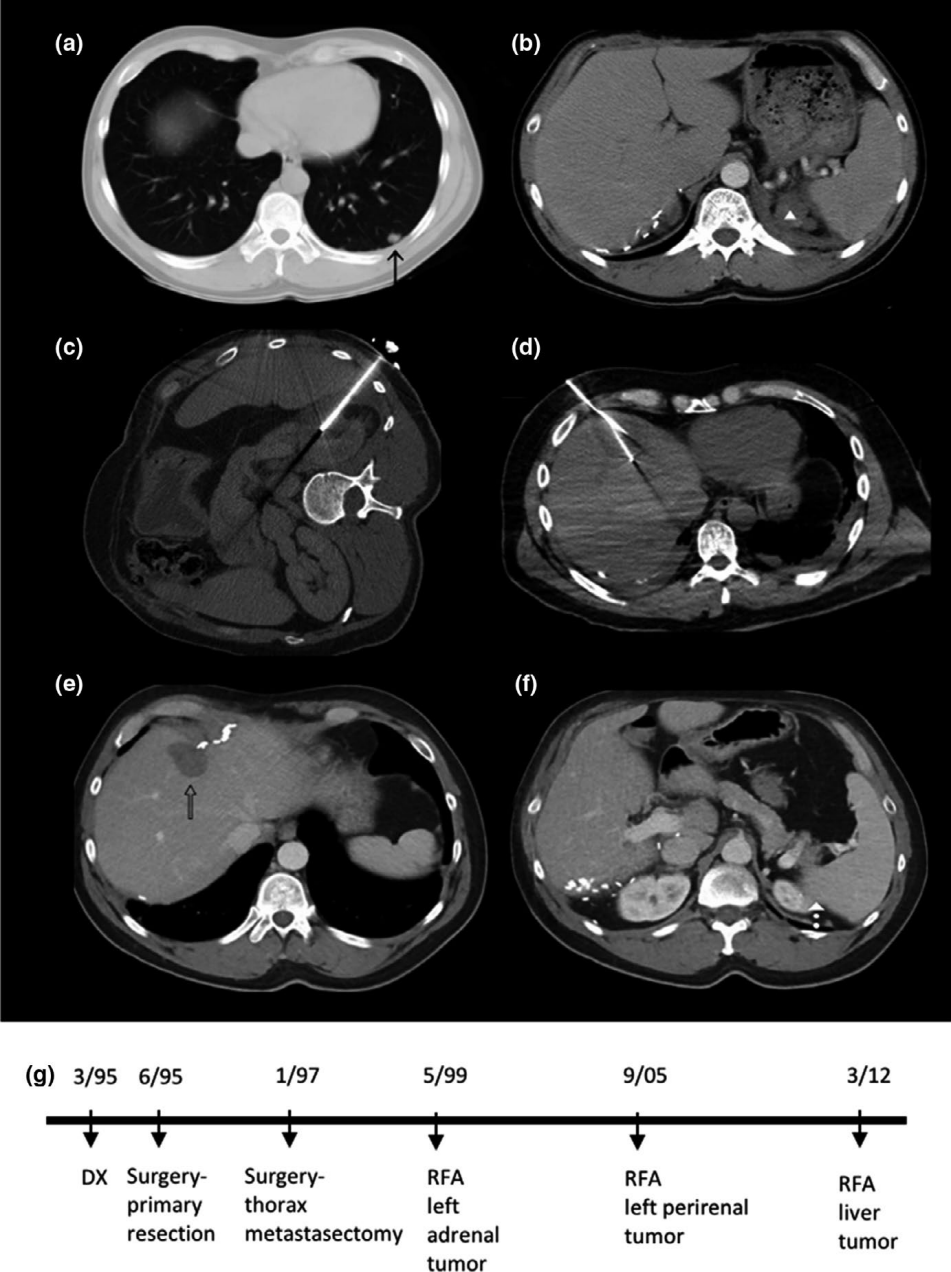
Case example. CT images of a patient that was diagnosed with stage IV adrenocortical carcinoma (ACC) and received his first image‐guided locoregional therapy (IGLT) 50 months after diagnosis. Contrast enhanced axial CT images pre‐ablation show lung metastasis (A, arrow) and left adrenal bed tumors (B, arrowhead). The patient was also treated with mitotane, systemic chemotherapy, and thoracic metastectomy. Three RFA sessions were performed at the left adrenal bed, left perirenal region, and segment 8 in the liver (intra‐procedural non‐contrast CT C, D) over a time span of 154 months. Long term surveillance with contrast enhanced axial CT 38 months following the last treatment session shows completely treated tumors (E, F). Timeline of interventions is provided in (G). This patient is alive 292 months following his initial diagnosis with no imageable active disease

Two patients suffered greater than or equal to CTC (Common Terminology Criteria version 5.0) grade 3 complications. One RFA session was complicated by an intrahepatic hematoma and the patient received PRBC transfusion. Another patient developed transient atrial fibrillation and electrolyte imbalance after a TAE. Three patients suffered grade 2 complications. One patient developed an abscess 2 months after paraspinal ablation, and was treated with percutaneous tube drainage and antibiotics. Two patients developed pneumothoraxes that required chest tube placement. Self‐limiting outcomes including pleuritic pain and transient elevations of liver function tests were not classified as complications. All patients received general anesthesia and were admitted to the hospital for the procedures. Standard PRN patient‐controlled analgesia and ketorolac were administered, per standard post‐ablation and post‐embolization protocols. An example of multimodality treatment paradigm associated with 292‐month survival in a 32‐year‐old patient with stage IV ACC at diagnosis is provided in Figure 1.

As of August 2019, 11 of the initial 39 patients were alive. For the surviving cohort, the median follow‐up interval from initial diagnosis was 169 months (range 63–292 months). For the 28 non‐surviving patients, median follow‐up time from initial diagnosis was 53 months (range 16–131 months). Two‐ and 5‐year OS rates for all included patients were, respectively, 84.5% and 51%.

**FIGURE 2:**
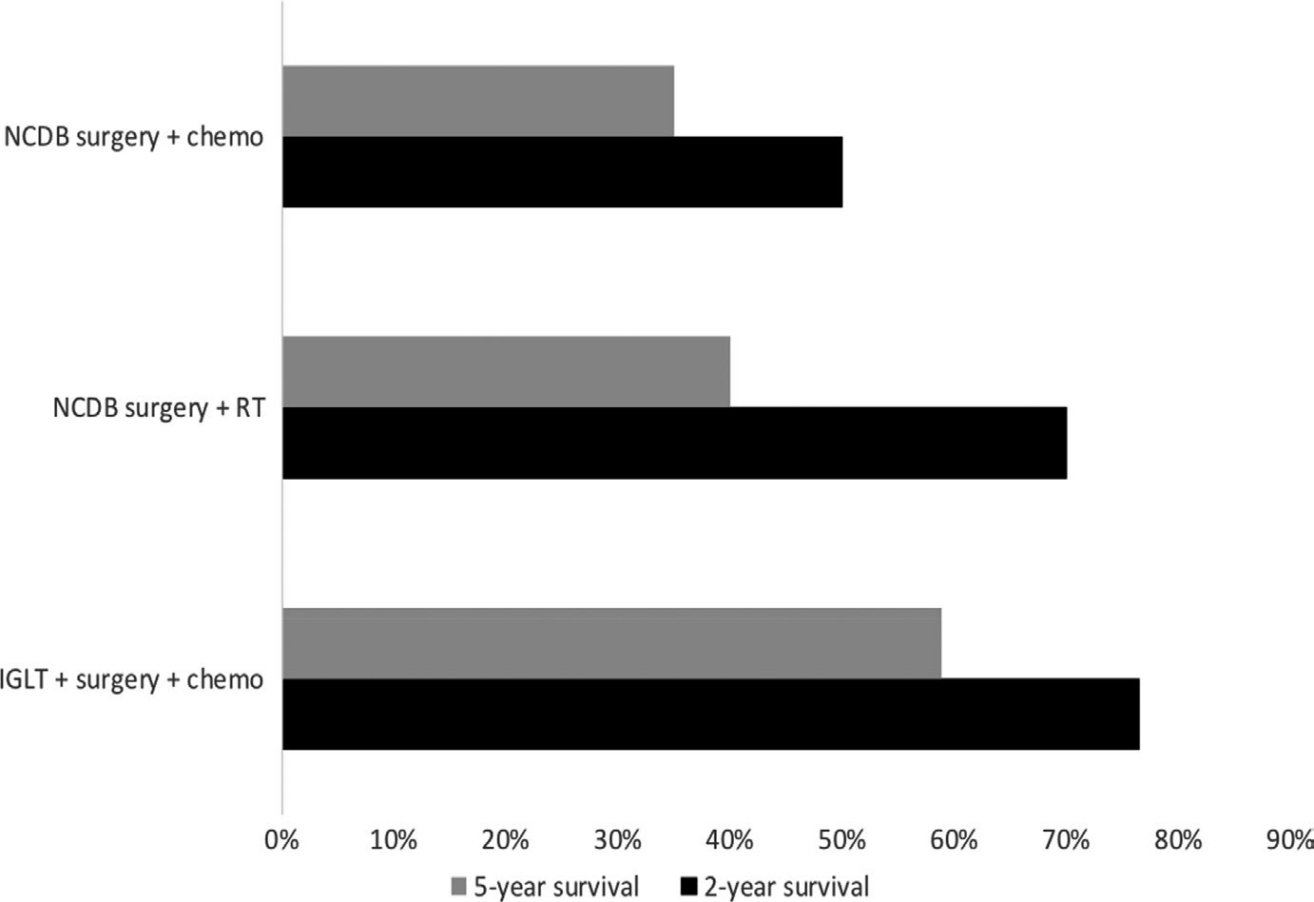
Subgroup survival analysis. Descriptive representation of the two‐ and five‐year survival rates of ACC patients in stage IV at diagnosis that were treated with primary resection and IGLT from our cohort (IGLT+surgery+chemo) and stage IV patients from the National Cancer Database (NCDB) which were treated with both surgery and chemotherapy or surgery and radiation therapy (RT)

Subgroup survival analysis was performed for the purpose of comparison with previously reported survival rates. Seventeen patients with stage IV disease at diagnosis were included, and survival rates were compared with the same stage group from the most recent NCDB report that was treated with surgical resection and adjuvant chemotherapy. Two‐ and 5‐year OS rates for this subpopulation were 76.5% and 59%, respectively. This survival compares favorably with 35% 5‐year survival of patients with metastatic ACC who were treated with adjunctive chemotherapy or radiation therapy (Figure 2).^18^

**FIGURE 3:**
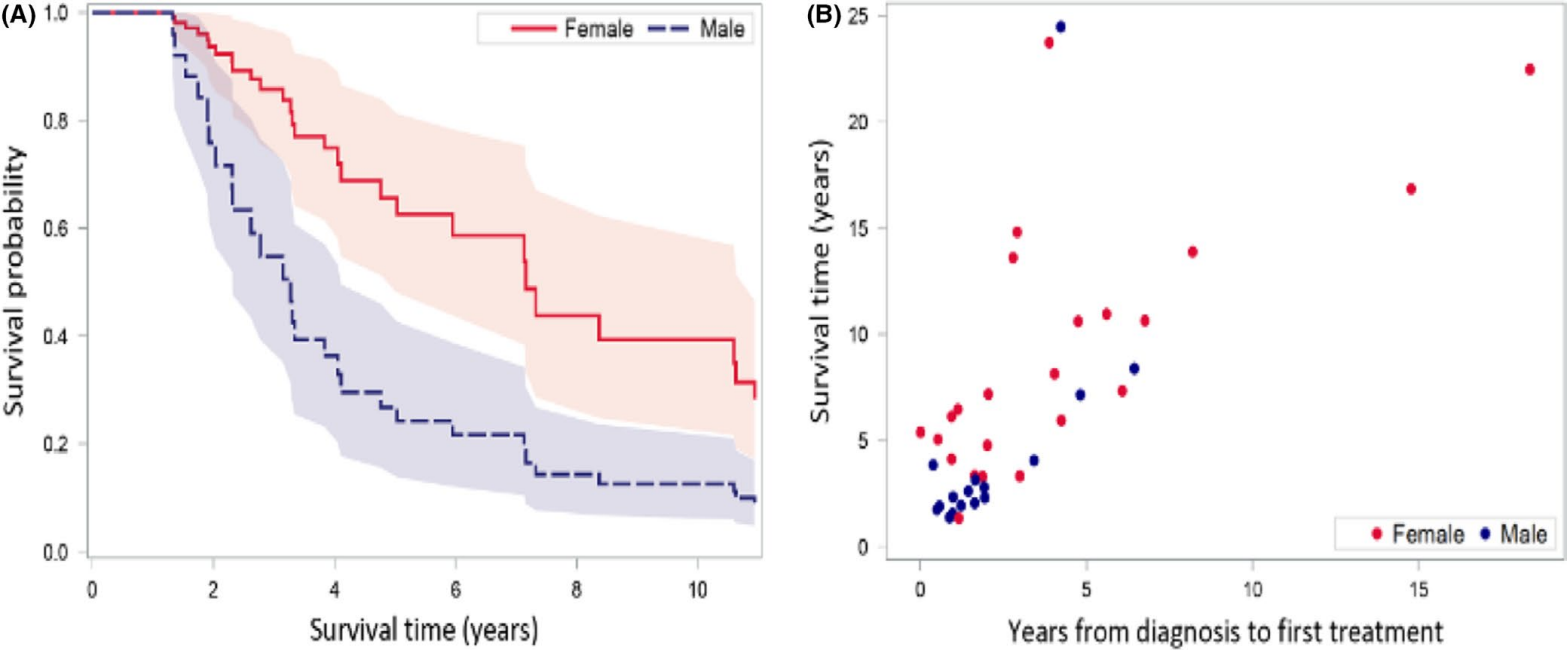
Prolonged survival predictors. Cox proportional hazards model found two variables to be predictors of prolonged survival: (A) female gender (*p* = 0.0004), and (B) longer time from diagnosis to first local treatment (*p* = 0.0012)

In our statistical analysis, two variables were found to be predictors of longer survival. One was gender (*p* < 0.001, Hazard Ratio = 0.23, 95% confidence Interval: 0.10–0.51): surprisingly, males had about four times the hazard rate of females (Figure 3A). Also predictive of longer survival was the time from diagnosis to the first IGLT (*p* = 0.001) (HR = 0.66, CI: 0.52–0.85), with each additional year between diagnosis and first IGLT, the hazard rate decreased by 34%, and the survival time increased (Figure 3B). Median time from diagnosis to first IGLT in our cohort was 21 months.

## 4 DISCUSSION

Similar to surgery performed to selectively remove sites of disease, IGLT offers the prospect of reducing disease burden in patients with advanced ACC to improve survival. Our patients had IGLTs added to standard treatments, and had associated overall 2‐ and 5‐year survivals that were better than those previously reported for patients with metastatic ACC.^2^, ^18^ IGLTs are well‐tolerated and safely applied with minimal morbidity. Comparison to natural historical controls, however, may only imply effect, and further corroborative experience is required to elevate the evidence and to validate these speculative associations.

Two potential predictors for prolonged survival were uncovered: female gender and longer time from initial diagnosis to first IGLT. Interestingly, gender has not been a significant factor in other studies predicting survival,^2^, ^20^ but conferred marked differences in the population reported here, with an uncertain causality. Increased time from diagnosis to first IGLT is a favorable prognostic factor in our cohort, and although subject to several variables, it may simply reflect the strong impact of a longer disease‐free interval on patient survival in ACC.^21^ Longer time from diagnosis to first IGLT may indicate favorable tumor biology, known as the “test of time” approach, and thus may represent a selection bias toward patients who theoretically have been preselected for their advantageous disease‐free interval. However, the disease‐free intervals reported here are comparable to those expected from ACC.^22^ It is possible that IGLT might “reset the clock” if it can meaningfully affect rapidly growing foci of metastatic deposits that might have otherwise been life‐limiting. We would note here that regardless of the explanation, the importance of longer disease‐free interval should not be dismissed. One can alternately view this as an indicator of who is most likely to benefit from IGLTs—in this case, those with longer disease‐free intervals where selective IGLT, much as a selective surgery, might prolong survival. This introduction of patient preselection bias is a major limitation.

The variable technical efficacy for complete treatment entered our statistical model, but after stepwise approach did not stay within the model, suggesting that patients might benefit from IGLT even if long‐term technical efficacy was not achieved.

Although optimal surgery by a surgical oncologist at the time of an initial diagnosis offers the best chance of OS for patients with ACC, outcomes following complete surgical resection of ACC are widely variable,^10^, ^23^ in part dependent upon the length of follow‐up data, and likely also upon patient selection. In addition, more than half of patients present with disease beyond the adrenal glands at diagnosis, often precluding complete surgical resection.^2^, ^3^ These patients have limited options for treatment, as systemic therapies have been associated with very modest improvements in progression‐free survivals. For a select group of these patients, it appears that there is a role for local extirpation and selective surgical resection, despite the impossibility of eradicating all sites of disease.^14^, ^24^ Repeated aggressive selective surgery has been shown to impact survival,^21^ and IGLTs may offer an extrapolation of this successful paradigm.

It has been proposed that in certain oligometastatic states, the elimination by ablation or surgery of all metastatic tumors may improve survival or, at times, even be curative, such as in certain breast, lung, and colon cancers.^25^, ^26^, ^27^ Supportive data for selective surgical approaches rely upon metastasectomy by resection, ablation, and/or stereotactic radiotherapy.^28^ Extrapolation of the above rationale in ACC provides support to an approach of selective surgical resection (by any means) for certain preselected patients with ACC. While surgery is of much value, it is difficult to perform repeated surgeries due to increased risks and prolonged recoveries. However, IGLTs can often be repeated, with both shorter interprocedural intervals and quicker recoveries.^12^ Additionally, IGLTs likely broaden the number of treatable patients, with an enhanced ability to reach tumors that are not amenable to surgical resection or radiation, due to technical factors, nearby critical anatomy, tumor volume, or multiplicity. Nonetheless, IGLTs have several limitations. Although ablations are conventionally limited to rather small lesions (less than 3 cm), our experience has shown success in much larger tumors.^11^ While most lesion locations would be amenable to ablation, some ablation sites, such as the dome of the liver, may be technically challenging, which introduces variability among centers. Transarterial and percutaneous therapies thus suffer from a lack of standardization. More rationale drug selection for transarterial delivery could impact outcomes for the treatment of ACC, but validation is challenging in such a rare disease. Low prevalence impairs randomized hypothesis‐driven studies.

There is minimal reported data on IGLTs in the management of ACC, and little to no long‐term data. Experience with percutaneous ablation has been described as a feasible approach, with outcomes at least comparable to those of local surgical resection.^11^, ^13^ Positive short‐term results are reported with both TAE and radioembolization.^29^, ^30^ When considering IGLTs, it is important to bear in mind that transarterial therapy is more often palliative in nature, while ablation is potentially extirpative and may be curative. Therefore, in situations in which the option of ablation or TACE exists, ablation may be more optimal, if a tumor‐free margin by imaging can be achieved.

This study has multiple limitations. The retrospective and non‐randomized experience of a single tertiary cancer center is reported. The study population is heterogeneous, and there is potentially major inherent selection bias toward the treatment of patients with more favorable tumor biology.

**FIGURE 4:**
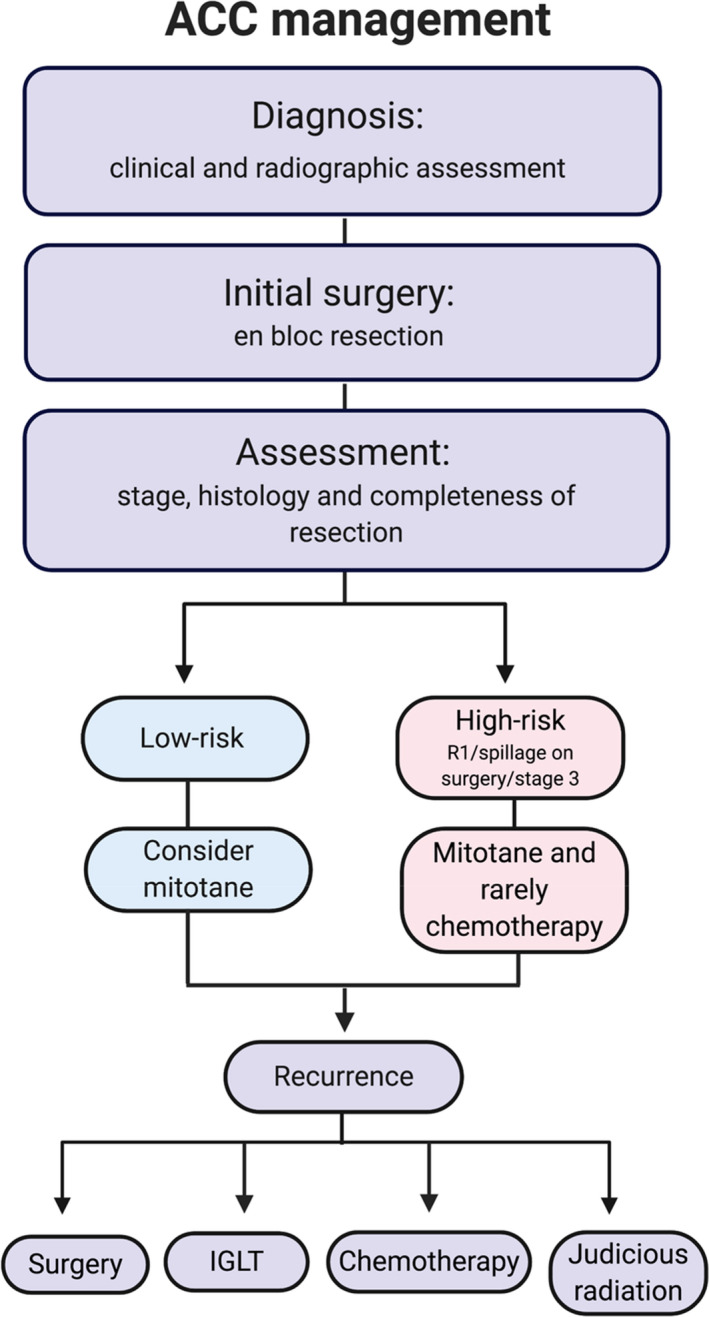
ACC management proposed algorithm

Our study includes inconsistent, sometimes limited imaging follow‐up. Methylation, chromosome alterations, and mutational profiles were not assessed, although these have recently been discovered to have prognostic value.^31^ Standardized response criteria for ablation are a surrogate for efficacy, but consensus guidelines for defining the efficacy of transarterial therapy for ACC are more subjective. Also, palliative and selective TACE are not performed for curative purposes. Despite these broad limitations, the reported experience characterizes the long‐term outcome of such an approach and suggests a meaningful role for IGLTs in the treatment of advanced ACC. This is particularly true for patients with longer time from diagnosis to recurrence, which probably preselects patients with favorable tumor biology. Although questions remain, these findings further support recently published guidelines from the European Society of Endocrinology, which also highlights the importance of considering local treatments for advanced ACC management.^5^ Finally, we would emphasize that in every case, we proceeded with the intention of completely eradicating the site of disease undergoing treatment. While our approach was selective in that at times not all lesions were treated, we never set out to eradicate only a portion of the selected lesion. Our proposed algorithm for the management of ACC is presented in Figure 4.

## 5 CONCLUSION

Treatment of patients with advanced ACC with IGLTs may be associated with prolonged survival, especially in females. Disease‐free interval is an understandable predictor of survival. Challenging clinical decisions for this rare disease with few effective treatment options may be informed by this two‐decade experience with IGLTs. Such a combined approach with surgery, ablation, and/or embolization may be associated with better than previously reported long‐term patient survivals.

## CONFLICT OF INTEREST

The authors have no conflict of interest to declare.

## Data Availability

The authors confirm that the data supporting the findings of this study are available within the article.
